# Plastic Compressed Collagen as a Novel Carrier for Expanded Human Corneal Endothelial Cells for Transplantation

**DOI:** 10.1371/journal.pone.0050993

**Published:** 2012-11-30

**Authors:** Hannah J. Levis, Gary S. L. Peh, Kah-Peng Toh, Rebekah Poh, Alex J. Shortt, Rosemary A. L. Drake, Jodhbir S. Mehta, Julie T. Daniels

**Affiliations:** 1 Department of Ocular Biology and Therapeutics, UCL Institute of Ophthalmology, London, United Kingdom; 2 Singapore Eye Research Institute, Singapore, Singapore; 3 Singapore National Eye Centre, Singapore, Singapore; 4 TAP Biosystems, Royston, Hertfordshire, United Kingdom; University of Missouri-Columbia, United States of America

## Abstract

Current treatments for reversible blindness caused by corneal endothelial cell failure involve replacing the failed endothelium with donor tissue using a one donor-one recipient strategy. Due to the increasing pressure of a worldwide donor cornea shortage there has been considerable interest in developing alternative strategies to treat endothelial disorders using expanded cell replacement therapy. Protocols have been developed which allow successful expansion of endothelial cells *in vitro* but this approach requires a supporting material that would allow easy transfer of cells to the recipient. We describe the first use of plastic compressed collagen as a highly effective, novel carrier for human corneal endothelial cells. A human corneal endothelial cell line and primary human corneal endothelial cells retained their characteristic cobblestone morphology and expression of tight junction protein ZO-1 and pump protein Na+/K+ ATPase α1 after culture on collagen constructs for up to 14 days. Additionally, ultrastructural analysis suggested a well-integrated endothelial layer with tightly opposed cells and apical microvilli. Plastic compressed collagen is a superior biomaterial in terms of its speed and ease of production and its ability to be manipulated in a clinically relevant manner without breakage. This method provides expanded endothelial cells with a substrate that could be suitable for transplantation allowing one donor cornea to potentially treat multiple patients.

## Introduction

The cornea is our transparent window to the world and its integrity and transparency are essential for proper functioning of the eye. The cornea is a highly organised tissue with three distinct cellular layers and two acellular layers. The acellular Descemet’s membrane separates the cellular stroma from the innermost endothelial layer, which is a monolayer of cells in direct contact with the aqueous humour of the anterior chamber. The corneal endothelial layer is responsible for the maintenance of corneal transparency by acting as a “leaky” barrier to allow nutrients to flow from the aqueous humour in the anterior chamber into the collagen stroma and then preventing swelling by actively pumping excess fluid out. This state of equilibrium is lost in disorders such as Fuchs endothelial dystrophy, which is characterised by a progressive oedema of the cornea, due to a loss of endothelial cell density. Fuchs is the most commonly occurring dystrophy in the US affecting approximately 4% of the population over the age of 40 [1]. For treatment of disorders such as Fuchs, several posterior lamellar techniques have been described as an alternative to the traditional full thickness corneal replacement known as penetrating keratoplasty (PK). These lamellar techniques replace only the defective endothelial layer and include Descemet’s stripping (automated) endothelial keratoplasty (DSEK (or DSAEK)) and Descemet’s membrane endothelial keratoplasty (DMEK). There are many advantages to the lamellar techniques over the PK procedure because the corneal surface is not compromised allowing for faster visual recovery, suture related problems are eliminated as endothelial keratoplasty requires no corneal sutures and wound healing complications are rare as the procedure can be performed through a self-sealing limbal or scleral tunnel incision at the periphery of the cornea [2], [3].

Although these new techniques are an improvement on the classic PK method, the worldwide donor cornea shortage is increasingly becoming an issue [4], compounded by the fact that demand for corneal transplantation is expected to increase due to a rise in the aging population globally [5]. This has led to considerable interest in the development of a strategy to treat endothelial disorders using cell replacement therapy as an alternative to one donor – one recipient tissue transplants. The considerable challenge here is that corneal endothelial cells are maintained in a G_1_ cell cycle phase arrested state and do not proliferate *in vivo* [6]. However, these cells do retain their proliferative capacity and many research groups have successfully stimulated cell division in order to expand endothelial cell numbers *in vitro* [7]–[10]. Expanded cell therapy could potentially allow many patients to be treated using one donor cornea and may alleviate some of the current donor shortage problems. However, this approach requires a supporting material with properties enabling easy transfer of the propagated cells to the recipient while at the same time exhibiting no detrimental effect on the functionality of the endothelial cell population [11].

**Figure 1:**
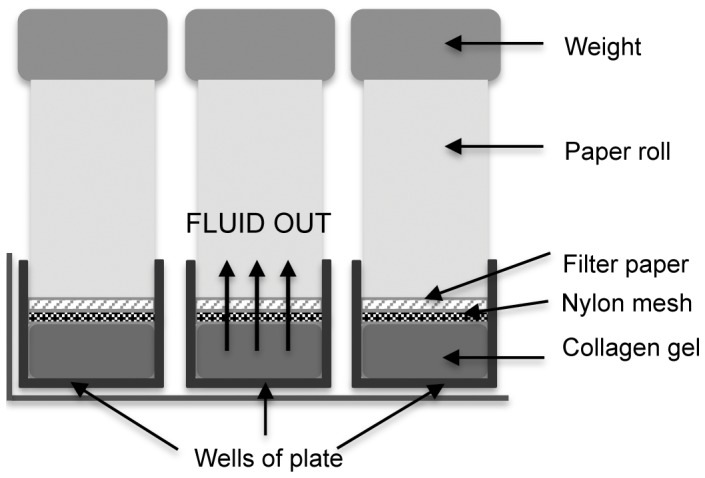
Plastic compression process. Schematic diagram showing the confined flow plastic compression process in a 12 well plate format to create RAFT.

**Figure 2:**
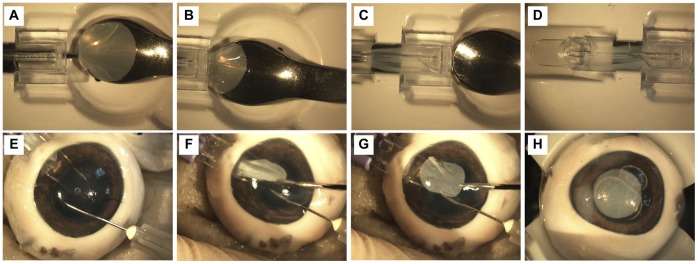
Loading and insertion of RAFT into an *ex vivo* porcine eye using Tan EndoGlide™. Representative photographs showing the process of loading (A–D) of the Tan EndoGlide™ with RAFT construct and insertion (E–H) of RAFT into the anterior chamber of an *ex vivo* porcine eye model. (A) Loading forceps grasp the edge of the RAFT construct from the spatula. (B) RAFT is pulled into the cassette and (C) automatically coils into a double coil configuration. (D) RAFT is fully loaded into the cassette with no upper surfaces touching. (E) Tan EndoGlide ™ is inserted into the anterior chamber that is prevented from collapsing using a column of saline via an inserted needle. (F) RAFT is pulled from the cassette (G) into the anterior chamber and positioned centrally before (H) an air bubble is inserted to appose RAFT to the posterior cornea.

We have developed a process of plastic compression of type 1 collagen hydrogels to produce a thin (60–200 µm) collagen membrane-like construct with enhanced mechanical properties, which we have termed Real Architecture For 3D Tissues (RAFT). If required, RAFT can be seeded directly with cells in the collagen before compression and we have previously shown it to be suitable for the generation of a human corneal epithelial cell surface layer [12]. The major advantage of RAFT is its rapid, simple and reproducible method of production and ability to be easily manipulated, particularly important if they are to be handled by surgeons for transplantation. Here we describe the first use of plastic compressed RAFT as a highly effective, novel carrier for cultured human corneal endothelial cells for transplantation.

## Materials and Methods

### Ethics Statement

All human tissue was handled according to the tenets of the Declaration of Helsinki and written consent was acquired from next of kin of all deceased donors regarding eye donation for research. This study was approved by the institutional review board of the Singapore Eye Research Institute/Singapore National Eye Centre.

### Donor Tissue

Cadaveric donor corneal rims with appropriate written research consent from next of kin were obtained from the Florida Lions Eye Bank (Miami, FL, USA). Three donor cornea pairs were used with donor age ranging from 15–24 years of age. Corneas were stored at 4°C in Optisol GS (Chiron Ophthalmics Inc., Irvine, California) and used within 13 days of enucleation.

### Isolation and Culture of Human Corneal Endothelial Cells

Human corneal endothelial cells (hCECs) were isolated using a 2-step peel and digest method as previously described [7]. Briefly, corneas were incubated in three washes of antibiotic/antimycotic solution in phosphate buffered saline (PBS; Life Technologies, Ltd., Paisley, UK) and then endothelial layer and Descemet’s membrane were removed for incubation with collagenase A (Sigma-Aldrich Ltd., Dorset, UK) for up to 6 hours at 37°C. Cell clusters were then subjected to TrypLE Express (Life Technologies, Ltd., Paisley, UK) treatment for 5 min at 37°C to disperse the cell clumps. Cells were then seeded onto fibronectin/collagen (FNC) coating mix (US Biologicals, MA, USA) coated 6 cm culture dishes (Falcon; BD Biosciences, Oxford, UK). Cells were cultured in human endothelial culture medium based on Engelmann’s F99 medium [13] with slight modifications as previously described [7]. Medium contained Ham’s F12:Medium 199 (1∶1), 5% foetal bovine serum, 10 ng/ml bFGF (all Life Technologies, Ltd., Paisley, UK), 20 µg/ml ascorbic acid, 20 µg/ml bovine insulin, 2.5 µg/ml transferrin and 0.6 ng/ml sodium selenite (all Sigma-Aldrich Ltd., Dorset, UK). Cell culture medium was changed every other day. Cells were sub-cultured after dissociation using TrypLE Express when confluent. Cells at passage 2 or 3 were seeded onto RAFT. Phase contrast images were taken to assess cell morphology using a Nikon TS100 microscope with a Nikon DS-FiI digital camera.

**Figure 3:**
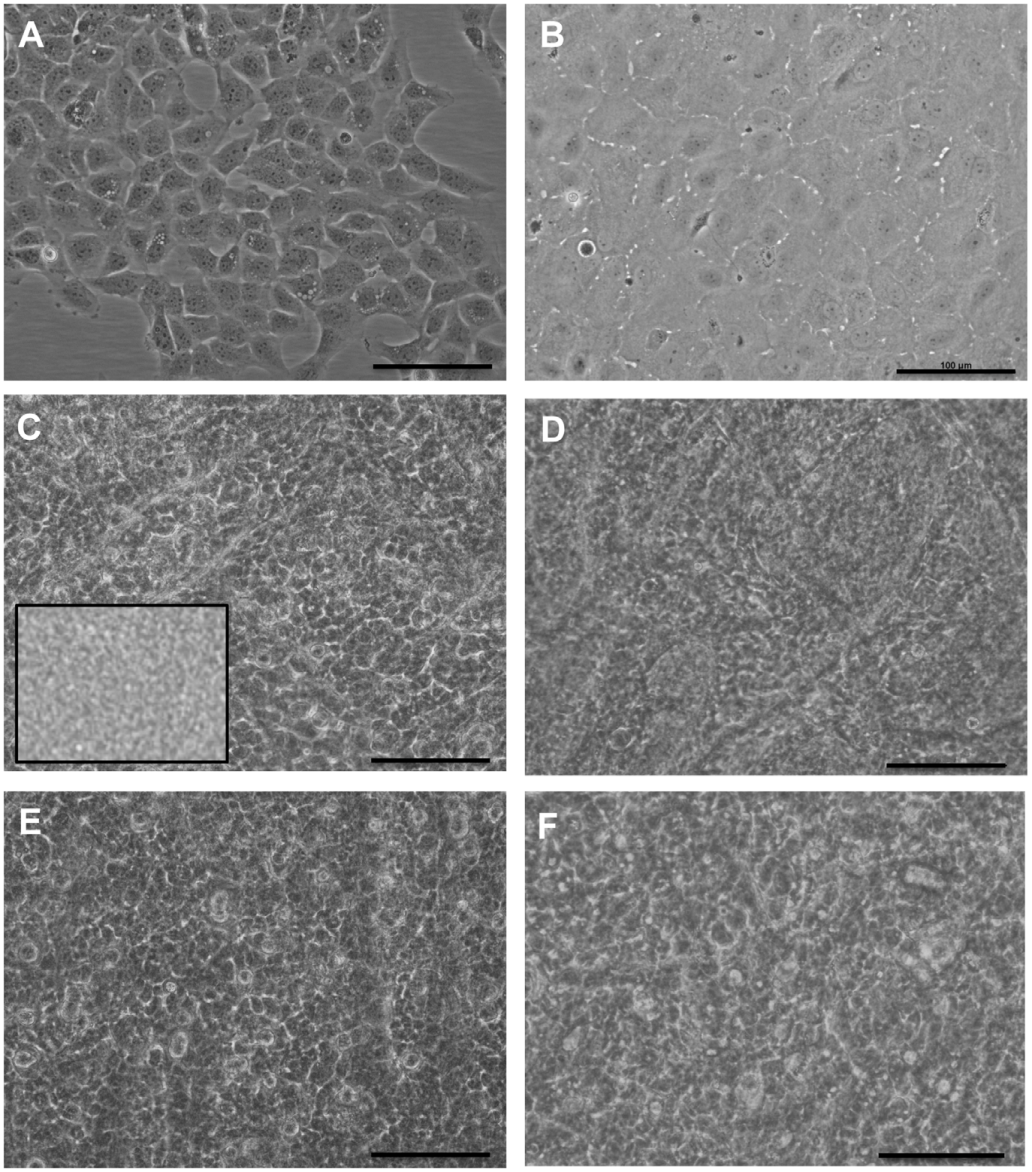
Morphology of cultured cells on RAFT. Phase contrast images of (A) hCECL and (B) primary hCECs on tissue culture plastic. Cells on RAFT, (C) hCECL seeded at a density of 2000 cells/mm^2^ (D) primary hCECs at 2000 cells/mm^2^ (E) hCECL at 3000 cells/mm^2^ and (F) primary hCECs at 3000 cells/mm^2^. Inset in (C) shows RAFT with no cells on the surface. Scale bars 100 µm.

**Figure 4:**
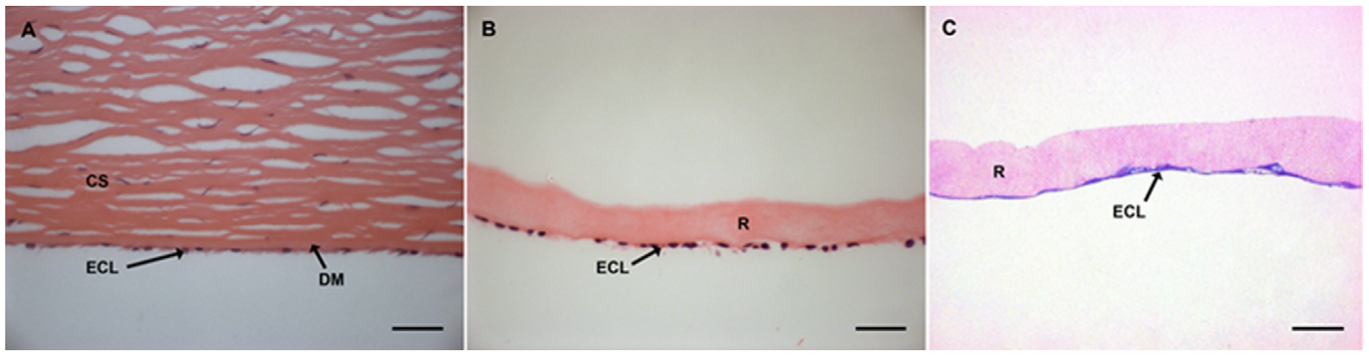
Comparison of conformation of cells in human cornea with cells on RAFT. Haematoxylin and eosin stained sections of (A) human corneal stroma (CS) and endothelial cell layer (ECL) separated by the Descemet’s membrane (DM), (B) hCECL (ECL) on RAFT (R) and (C) toluidine blue and fuschin stained primary hCECs (ECL) on RAFT (R). Scale bars 50 µm.

### Culture of the Human Corneal Endothelial Cell Line

A human corneal endothelial cell line (hCECL) was cultured as per supplier’s instructions (B4G12; DSMZ, Germany). Cells were seeded onto chondroitin sulphate and laminin (CS/L; both Sigma-Aldrich Ltd., Dorset, UK) coated dishes (Corning Life Sciences, Amsterdam, Netherlands) in culture medium consisting of human endothelial-SFM (Life Technologies, Ltd., Paisley, UK) supplemented with 10 ng/ml bFGF (Sigma-Aldrich Ltd., Dorset, UK). Cell culture medium was changed every 2 days and cells passaged using 0.05% trypsin solution (Life Technologies, Ltd., Paisley, UK) before reaching confluence. Trypsin was neutralised using protease inhibitor cocktail (Roche Diagnostics, West Sussex, UK) and cells seeded at 2000 cells/mm^2^.

### Preparation of Collagen Solution

Collagen gels were prepared by sodium hydroxide (Sigma Aldrich, Dorset, UK) neutralization of a solution that finally comprised 80% vol/vol sterile rat-tail type I collagen (2.06 mg ml-^1^; First Link, Birmingham, UK) and 10% vol/vol 10x Minimum Essential Medium (Life Technologies, Ltd., Paisley, UK). After neutralisation, the final 10% vol/vol hCEC medium was added. This solution was then left on ice for 30 min to prevent gelling while allowing dispersion of any small bubbles within the solution before casting in well plates.

### Plastic Compression of Collagen Gels

Collagen gels were plastic compressed using a confined flow compression method. A volume of 2.2 ml of collagen solution was added to each well of a 12 well plate (Nunc; Fisher, Loughborough, UK). Well plates were incubated at 37°C for 30 min to allow the collagen to undergo fibrillogenesis. Once the gels were set they were subjected to a confined compression (Fig. 1). Briefly, a sterile nylon mesh and a sterile filter paper circle were placed directly on top of a collagen gel and then a chromatography paper roll was added. A 35 g load was then applied to the system for 15 min to allow compression of the collagen gel with loss of fluid in a confined, upward flow direction through the paper roll. This process yielded a thin collagen construct, that we have termed RAFT, which was then either kept in place in a 12 well plate for hCECL culture or trephined using a 8.25 mm trephine (Coronet, Network Medical Products Ltd., Ripon, UK) to obtain small discs for hCEC culture. The trephined discs were transferred to an organ culture dish (Falcon; BD Biosciences, Oxford, UK) and maintained in a small amount of PBS until cell seeding. The thickness of representative RAFT constructs was then measured using optical coherence tomography (OCT) with an anterior segment lens (Spectralis, Heidelberg Engineering, Hemel Hempstead, UK). Thickness was measured at 3 positions along the length of a scanned area in the centre of each of three replicate constructs.

**Figure 5:**
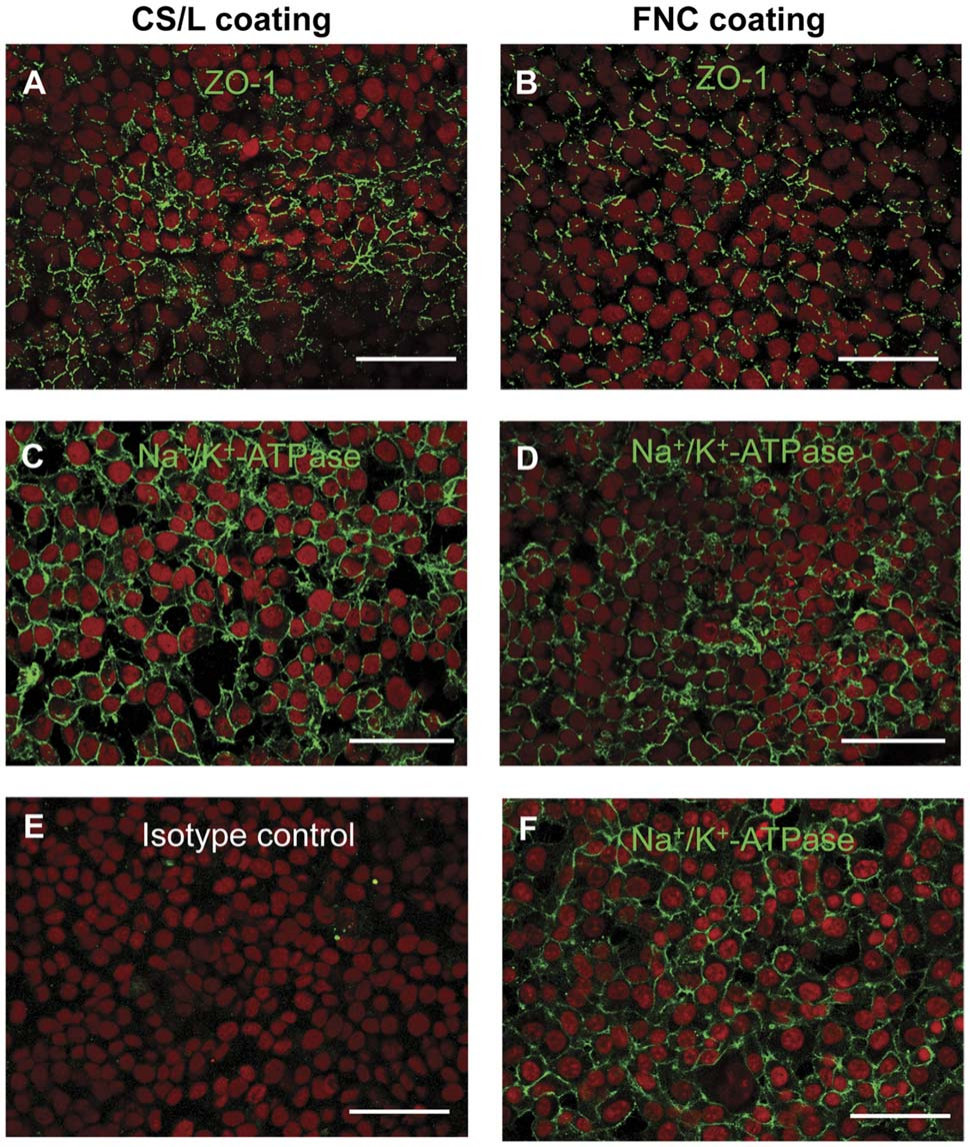
Immunochemical analysis of hCECL cells on RAFT. hCECL seeded on RAFT (A) at 3000 cells/mm^2^ with C/L coating or (B) 3000 cells/mm^2^ on FNC coating stained with ZO-1 (green) and counterstained with propidium iodide (red). hCECL seeded on RAFT (C) at 2000 cells/mm^2^ with C/L coating or (D) seeded at 4000 cells/mm^2^ on FNC coating stained with Na^+^/K^+^-ATPase (green) and counterstained with propidium iodide. (E) Negative isotype control. (F) hCECL on permanox slides with FNC coating stained with Na^+^/K^+^-ATPase (green) and counterstained with propidium iodide. Scale bars 50 µm.

**Figure 6:**
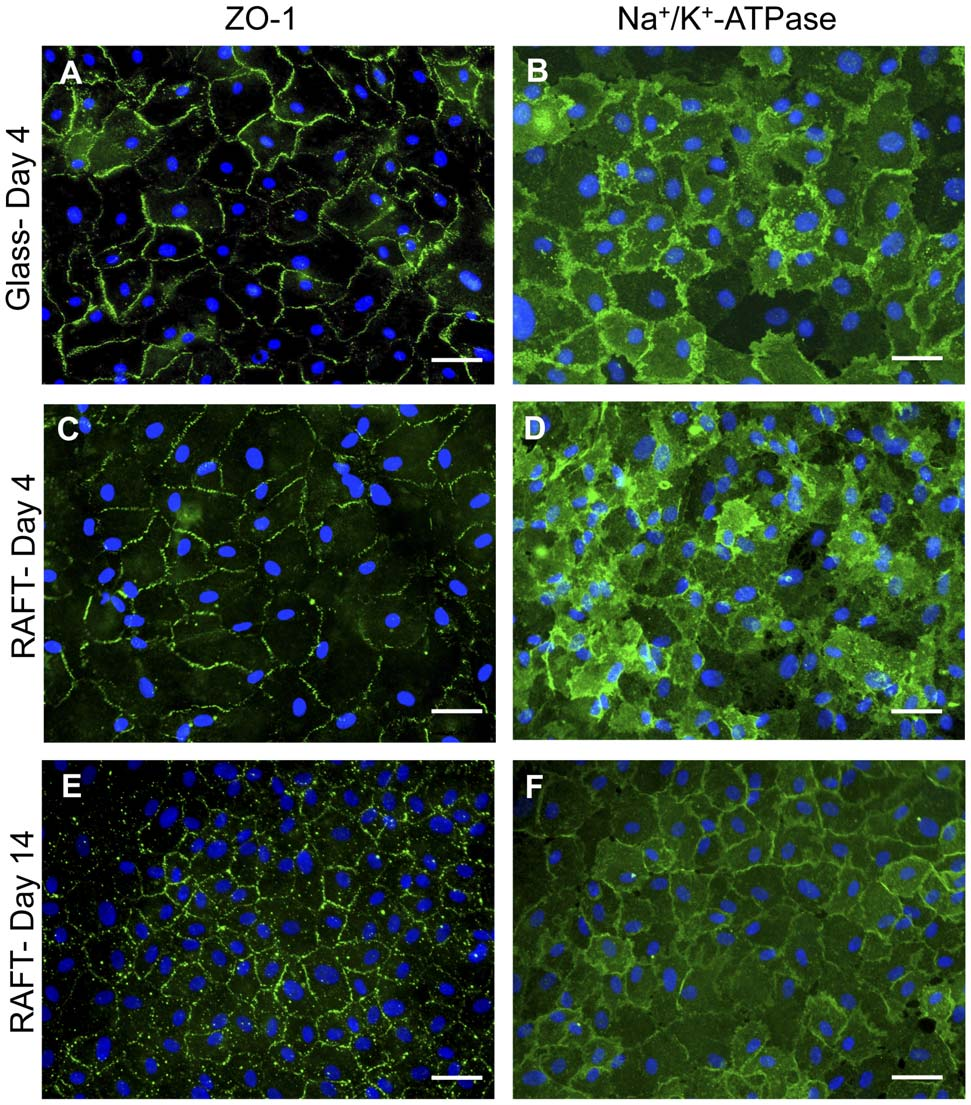
Immunochemical analysis of primary hCECs on RAFT. Primary hCECs seeded on glass slides and fixed after 4 days, stained with (A) ZO-1 or (B) Na^+^/K^+^-ATPase (green) counterstained with DAPI (blue). Primary hCECs seeded onto RAFT stained with either (C and E) ZO-1, (D and F) Na^+^/K^+^-ATPase (green) counterstained with DAPI (blue). (C and D) fixed after 4 days in culture or (E and F) after 14 days. Scale bars 50 µm.

### Ease of Handling of RAFT for Transplantation

Porcine whole globes were obtained from First Link Ltd., Birmingham, UK. Excess tissue was dissected from the scleral globe to clean the eyes. Acellular RAFT constructs were prepared as above and 8.25 mm discs were trephined. A RAFT disc was placed onto the donor well of the Tan EndoGlide™ preparation base using a metal spatula. RAFT was then pulled into the cartridge using loading forceps as per manufacturer’s instructions. A 4 mm wound was made in the sclera to allow insertion of the Tan EndoGlide™ and a small incision made on the opposing side to allow insertion of forceps. The anterior chamber was maintained by attachment of an infusion of saline at a height of 40 cm via a 28-gauge needle through the cornea into the anterior chamber. The RAFT construct was then pulled from the cassette into the anterior chamber using a pull- through technique. Once free floating in the anterior chamber, a bubble of air was introduced behind the RAFT construct to maintain its apposition with the posterior stroma. Representative photographs were taken of the loading and insertion procedures as seen through a dissecting microscope, (SMZ1500, Nikon, Kingston upon Thames, UK).

### Seeding of Endothelial Cells onto RAFT

RAFT constructs were coated with either FNC coating mix or CS/L and then hCECLs were seeded onto the surface in 12 well plates at a density of 2000–4000 cells/mm^2^ in a volume of 2 ml medium. Primary hCECs were seeded onto FNC coated RAFT discs in organ culture dishes at a density of 2000–3000 cells/mm^2^ in a volume of 20 µl. Several hours later, after cells had attached, wells were flooded with endothelial cell culture medium. Dishes were placed in an incubator at 37°C with 5% CO_2_ in air_._ Cell culture medium was changed every other day and constructs fixed for staining on day 4 or day 14 for longer-term cultures.

### Histological Staining of Paraffin Embedded Sections

Human central corneal specimens and RAFT constructs with hCECL on the surface were fixed for 30 min with 4% PFA before processing for paraffin embedding. Tissue sections (6 µm) were cut on a microtome, rehydrated through alcohols to water, stained with haematoxylin and eosin, mounted and coverslipped with DPX. Sections were imaged using a Zeiss 510 Microscope and Axiovison software.

**Figure 7:**
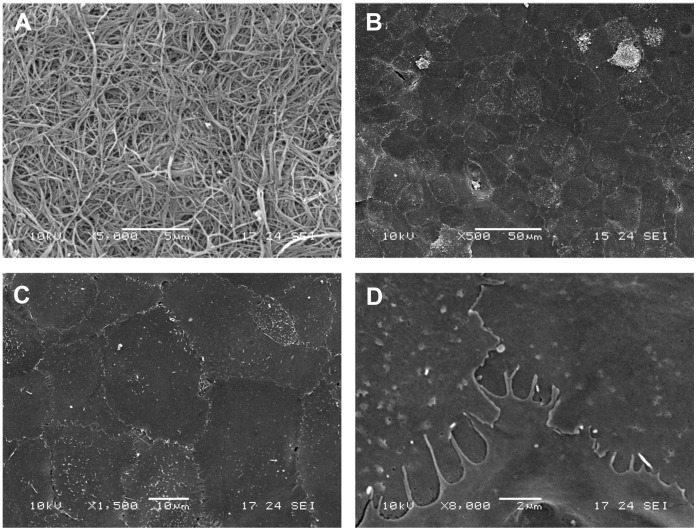
Scanning electron microscope characterisation of RAFT with hCECs. (A) Representative micrograph of the surface of RAFT constructs. (B) Representative low magnification SEM image showing confluent monolayer of hCECs on RAFT. (C) Higher magnification image showing cell borders between cells and (D) at high magnification over-lapping finger-like projections onto juxtaposed cells. Scale bars A 5 µm, B 50 µm, C 10 µm, D 2 µm.

**Figure 8:**
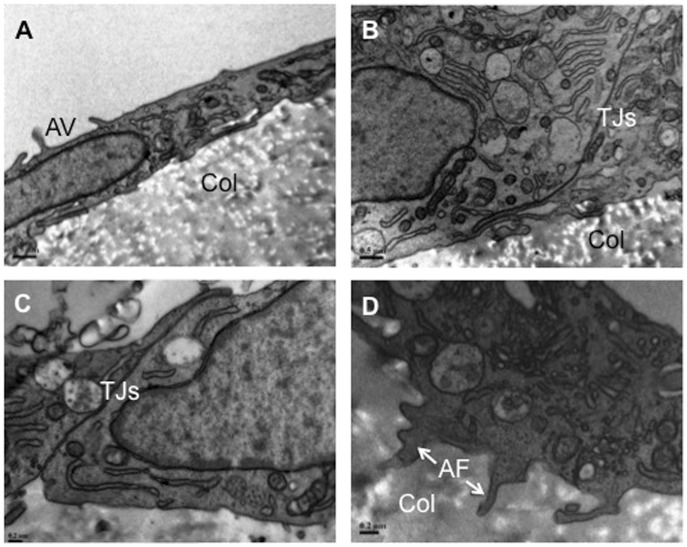
Transmission electron microscope characterisation of hCECs on RAFT. (A) Representative image showing apical microvilli (AV) on the endothelial surface of cells attached to collagen RAFT (Col). (B) Representative image showing tight junctions (TJs) between adjacent cells on collagen RAFT (Col). (C) Further evidence of tight junctions (TJs) at higher magnification. (D) Anchoring filaments (AF) from the overlying endothelium are seen extending into the collagen substrate (Col). Scale bars A, B 0.5 µm, C, D 0.2 µm.

### Immunostaining of Endothelial Cells on RAFT

RAFT constructs with hCECL and hCECs on the surface or hCECL and hCECs cultured on permanox or glass slides respectively were fixed with 100% ice-cold ethanol on day 4 or day 14 (hCECs). Constructs were then blocked in 10% normal goat serum for 30 min before addition of the primary antibody in blocking buffer for 1 hour at room temperature. Mouse anti-human ZO-1 (IgG1, 1∶50; BD Biosciences, Oxford, UK) and mouse monoclonal Na^+^K^+^ adenosine triphosphatase (ATPase; IgG1, 1∶40; Santa Cruz, Biotechnology, Heidelberg, Germany) were used. Negative controls were cells incubated with an anti-mouse IgG1 isotype control (Biolegend UK Ltd., Cambridge, UK). After washing in PBS, secondary antibody Alexa Fluor 488 (1∶750; Life Technologies, Ltd., Paisley, UK) was incubated with constructs at room temperature for 30 min. Constructs were then mounted on slides with vectorshield mounting medium with 4′,6-diamidino-2-phenylindole (DAPI; Vector Labs, Peterborough, UK) or propidium iodide (PI, Sigma-Aldrich Ltd., Dorset, UK) and sealed with nail varnish for imaging on a Zeiss Imager microscope with Axiovision software (hCECs) or a Zeiss LSM 510 confocal microscope (hCECL).

### Endothelial Cell Density and Cell Size on RAFT

Cell density of hCECs was measured by counting cell numbers in at least 4 fields of view from 4 different RAFT constructs seeded with cells. The number of cells per mm^2^ was then calculated. The average size of hCECs and hCECL cells was calculated by taking the area of field of view and dividing by average cell number per field to determine approximate cell area in µm^2^± SEM. An unpaired *t*-test was performed to determine statistical significance with values deemed to be significant if *p*<0.05.

### Electron Microscopy Analysis of Endothelial Cells on RAFT

RAFT constructs were examined using transmission electron microscopy (TEM). RAFT constructs were fixed with 2% paraformaldehyde and 2% glutaraldehyde in 0.1 M sodium cacodylate buffer, pH 7.4 (Electron Microscopy Sciences (EMS), Hatfield, PA, USA) at 4°C overnight. Constructs were then washed in sodium cacodylate buffer and post-fixed in 1% osmium tetroxide and potassium ferrocyanide (EMS) to enhance membrane contrast. After extensive rinsing with distilled water, tissues were dehydrated in a graded series of ethanol, and embedded in Araldite (EMS). Semi-thin sections of 0.5–1 µm were cut with a Reichert-Jung Ultracut E Ultramicrotome (C. Reichert Optische Werke AG, Vienna, Austria), counterstained with toluidine blue/basic fuchsin and examined using an Axioplan, Zeiss light microscope (Carl Zeiss, Germany). The ultra-thin sections of 60–80 nm thickness were cut and collected on copper grids, double stained with uranyl acetate and lead citrate for 20 min each, then viewed and imaged at 100 kV on a Philips EM 2085 transmission electron microscope (FEI Electron Optics BV, Eindoven, Netherlands). For scanning electron microscopy (SEM), specimens were immersed in a fixative containing 2% glutaraldehyde, 2% paraformaldehyde and 0.1 M sodium cacodylate (pH 7.4) overnight at 4°C. They were then transferred and stored in sodium cacodylate buffer (EMS). Before processing, the samples were washed twice in distilled water and were secondary fixed in 1% osmium tetroxide (FMB, Singapore), for 2 hours at room temperature. Following this, samples were dehydrated, critical point dried (Bal-Tec, Liechtenstein, Germany) and mounted on SEM stubs using carbon adhesive tabs and finally sputter coated with a thin layer of gold (10 nm; Bal-Tec). Samples were imaged with a field-emission SEM (XL 30 FEG SEM; FEI Company/Philips, Eindoven, The Netherlands) at 10 kV.

## Results

### Ease of Handling of RAFT for Transplantation

Acellular RAFT constructs were created and trephined into 8.25 mm discs. To demonstrate ease of handling of RAFT for transplantation, we used a Tan EndoGlide™ insertion system that is used clinically to deliver DMEK or DSEK tissue to the anterior chamber. RAFT could be successfully loaded into the Tan EndoGlide™ system (Fig. 2A–C), curling inwards in the intended manner that would protect the endothelial layer as it does for DMEK (Fig. 2D). An *ex vivo* porcine eye model was used to confirm that RAFT could be successfully delivered from the Tan EndoGlide™ to the anterior chamber through a typical 4 mm scleral wound using a pull-through technique (Fig. 2E–G). After removal of all instruments and injection of an air bubble to position RAFT apposed to the posterior stroma, it is possible to see that RAFT remains fully intact with no signs of tearing after the full surgical procedure (Fig. 2H), suggesting the material has suitable mechanical properties to enable transplantation.

### Culture of Human Endothelial Cells on RAFT

RAFT thickness before cell seeding was assessed using OCT and found to be on average 74.1±2.04 µm (mean± SD). The morphology of endothelial cells on tissue culture plastic and on the surface of RAFT was then assessed using light microscopy. The hCECL grew in strict monolayer formation comprising small polygonal cells when cultured on CS/L coated tissue culture plastic (Fig. 3A). hCECs expanded and then passaged (up to passage 3) on FNC coated tissue culture plastic displayed a polygonal morphology typical of human corneal endothelium (Fig. 3B). hCECL and hCECs were seeded at varying densities onto RAFT to determine the optimum seeding density to produce a confluent monolayer. The background topology of acellular RAFT caused some interference with cell image capture (Fig. 3C inset). However, on closer inspection and in comparison to acellular constructs, cell morphology could still be discerned. When seeding either hCECL (Fig. 3C) or hCECs (Fig. 3D) at 2000 cells/mm^2^, cells attached within hours and after 24 hours had formed a monolayer on the RAFT surface. The same was true for both hCECL (Fig. 3E) and hCECs (Fig. 3F) seeded at 3000 cells/mm^2^ and there was no discernable difference between the cells at this 24-hour time point. The final cell density of hCECs after 4 days culture on RAFT seeded at a density of 2000 cells/mm^2^ was calculated and found to be on average 1941.2 cells/mm^2^.

### Conformation of Endothelial Cell Layers on RAFT

Tissue sections of human cornea and endothelial cells on RAFT were stained with haematoxylin and eosin or toluidine blue and basic fuschin to reveal similarities in conformation of the cells on RAFT compared to native tissue. Human endothelial cells line the posterior portion of the cornea in a single monolayer separated from the collagen stroma by the Descemet’s membrane (Fig. 4A). hCECL on RAFT form a confluent monolayer on the collagen surface (Fig. 4B) that is very similar to the formation seen in corneal specimens. The same can be said for hCECs seeded onto RAFT where an endothelial monolayer can be seen (Fig. 4C).

### Endothelial Cell Marker Expression of Cells on RAFT

The effect of cell density and surface coating on the expression of ZO-1 and Na^+^ K^+^ -ATPase was tested using the hCECL. No apparent differences were seen in the pattern of ZO-1 expression in cells seeded onto RAFT at 3000 cells/mm^2^ on CS/L coating compared with FNC coating (Fig. 5A and B, respectively). Equally, cells seeded at different concentrations, 2000 compared with 4000 cells/mm^2^, showed comparable expression patterns of Na^+^/K^+^-ATPase (Fig. 5C and D, respectively). This consistent pattern of high expression at cell borders of cells on RAFT also matched that of cells on permanox slides coated with FNC coating (Fig. 5F), whereas representative negative isotype controls showed no staining in these areas (Fig. 5E). The average cell area of hCECs on RAFT was 614.8±157.6 µm^2^ compared with 185.1±14.5 µm^2^ for hCECL cells, which was a significant difference (*p*<0.05). The large variability in cell size observed with hCECs is fairly typical of cells in primary cultures compared to the often homogenous cell lines. In both cases hCECs expressed ZO-1 (Fig. 6A and C) and Na^+^/K^+^-ATPase (Fig. 6B and D) in abundance whether on glass (Fig. 6A and B) or RAFT (Fig. 6C and D) after 4 days in culture. This expression pattern was also seen after 14 days (Fig. 6E and F), indicating that cells remain viable and maintain their typical endothelial phenotype even after longer- term culture. Additionally, the expression pattern of ZO-1 confirmed the typical cobblestone appearance of hCECs on RAFT by labelling cell junctions and borders. The isotype matched controls were negative as expected (data not shown).

### Ultrastuctural Properties of Endothelial Cells on RAFT

Scanning electron microscopy revealed the randomly orientated collagen fibre structure of the seeding surface of RAFT (Fig. 7A). A low magnification image of hCECs on the collagen surface showed a confluent monolayer of polygonal cells (Fig. 7B) and at higher magnification the tightly opposed borders were apparent (Fig. 7C), additional evidence of typical endothelial morphology. Further magnification reveals the highly interdigitated apical flaps of cell borders and microvilli on the cell surface (Fig. 7D). Transmission electron microscopy confirmed apical microvilli on the cell surface, which leads to significant enlargement of cell surface area (Fig. 8A) and tight junctions between adjacent cells (Fig. 8B and C). Anchoring filaments protruding into RAFT were also identified, which presumably aid attachment and security of the endothelial layer to the underlying collagen substrate (Fig. 8D).

## Discussion

The application of plastic compressed collagen for the culture of human corneal endothelial cell layers offers an attractive alternative for surgical restoration of corneal endothelium using a simple and rapidly produced tissue engineered substrate. The present study demonstrates that RAFT is a suitable substrate for culture of corneal endothelial cells and additionally indicates that this carrier has sufficient mechanical strength for transplantation using current clinical delivery techniques. This feasibility study provides encouraging evidence for further development using an *in vivo* model to confirm endothelial functionality and RAFT suitability in a living system.

Corneal endothelial cell loss or damage leads to stromal oedema, loss of transparency, and will eventually lead to blindness. The transplantation of a healthy endothelial layer is generally required to reverse the oedema. The worldwide shortage of donor corneal tissue has led to increased pressure to optimise protocols for the reproducible expansion of endothelial cells *in vitro* to enable development of tissue engineered endothelial constructs. Published methods for isolation and expansion of endothelial cells vary greatly between research groups [5], but success is difficult to evaluate when donor variability is not taken into consideration. Colleagues have evaluated the effectiveness of published culture methods comparing the efficiency in supporting endothelial cell growth from a single donor to eliminate the differences caused by donor sample variability [7]. They compared four different cell culture media after isolating cells using a two-step peel and digest method and were able to determine the optimal culture medium for expansion of cells. Endothelial cells were cultured for this study using this method and it was found that cells could be expanded from an initial starting number of approximately 3×10^5^ per donor pair to between 6–15×10^6^ cells after only 2 passages (GS Peh and JS Mehta, unpublished observation). In this study we were able to seed human corneal endothelial cells at a concentration that produced a final density of, on average, 1941.2 cells/mm^2^. This figure is within range of the 2000 cells/mm^2^ minimum for transplantation which is employed by 70% of European eye banks, according to a 2010 European Eye Bank Association Directory report [14]. Seeding at these densities with the number of cells obtained from one cornea and estimating conservatively, this could be equated to 20 RAFT preparations, 10 mm diameter in size, meaning that one donor cornea pair could potentially treat 20 eyes, a dramatic improvement on the one to one approach with whole tissue transplant.

The endothelial layer is at the boundary of the fluid filled anterior chamber and a major function of the corneal endothelium is to maintain corneal transparency by regulating corneal hydration. The “leaky” barrier formed by the endothelial cells allows aqueous humour to flow into the cornea to supply nutrients to the avascular stroma and tight junctional proteins such as ZO-1, expressed at lateral cell borders, are thought to regulate paracellular permeability. The tendency for stromal swelling is counteracted by the removal of excess fluid via Na^+^ K^+^ -ATPase ionic pumps located on the lateral plasma membranes. Hence, the expression of ZO-1 and Na^+^ K^+^ -ATPase, detected by antibodies, in primary endothelial cells and endothelial cell lines in culture supports the assumption that cells are exhibiting proper pump function as seen in native tissue. Tissue engineering an endothelial sheet *ex vivo* relies upon the fact that the cells can be expanded *in vitro*, but this approach would fail if cells did not survive or lost functional phenotype, i.e. expression of ZO-1 or Na^+^ K^+^ -ATPase, during the time taken to expand to confluence or after deposition on a carrier substrate. The culture of primary endothelial cells *in vitro* is a considerable challenge, requiring specific knowledge and skills and donor material of a particular specification. For initial exploratory experiments to determine the suitability of RAFT as a carrier for endothelial cells, a human corneal endothelial cell line was used before use of primary endothelial cells. We have shown that both the hCECL cells and primary hCECs seeded onto RAFT attach and mature to form a stable confluent monolayer after only 4 days in culture. Cells retained the typical characteristics of endothelial cells including cobblestone morphology and ultrastructural features of apical microvilli and tight junctions between neighbouring cells and even after 14 days were shown to retain expression of ZO-1 and Na^+^ K^+^ -ATPase. This suggests that RAFT is a suitable substrate for long-term culture of human endothelial cells for subsequent transplantation. Additionally, this validates the use of the endothelial cell line as an experimental alternative when it is not possible to culture primary cells due to lack of suitable donor material or knowledge of the complex culture protocols. A simple corneal endothelial tissue equivalent suitable for many *in vitro* testing applications can be rapidly created using the endothelial cell line with RAFT as the stromal portion.

A number of different cell carriers have been trialled for the purpose of endothelial layer construction but the possibilities are limited by the specific requirements of a substrate in this context. The required properties include; cytocompatibility, reproducibility, ease of production/supply, transparency, ability to be handled easily by surgeons ideally with tuneable properties such as thickness. Amongst the materials tested by others are bioengineered materials such as collagen vitrigels [15], atellocollagen and gelatin hydrogel sheets [16], silk fibroin [17], and tissues such as the xenogeneic substrate of bovine corneal posterior lamellae [18], human anterior lens capsule [19] and amniotic membrane [20]. Tissues such as amniotic membrane are beneficial, as they have been widely used in ocular surgery and have already been proven to successfully support the culture of other ocular cells such as limbal epithelial cells ([21]–[24] and reviewed in [25]). However, the donor variability between biological materials such as these renders them unreliable and amniotic membrane in particular displays sub-optimal transparency limiting its use in this context. An *in vivo* study using RAFT would provide important information regarding degradation time in the presence of cells and anterior chamber fluids as well as the effect of a functional endothelial layer on RAFT transparency. Bioengineering a material is advantageous as variability is limited and materials can be selected based on their desirable properties. However, the gelatin and collagen hydrogels and silk fibroin mats which have been trialled in this area lack mechanical strength required for surgical use and can be very fragile upon handling. Collagen vitrigels are also not ideal as there is a relatively lengthy process involved in the production of these materials (reviewed in [26]). The crucial advantage of our RAFT biomaterial is the simple and rapid method of production, which yields multiple reproducible constructs with limited variability between batches. Additional advantages of the process are that the properties of the material are tuneable allowing the user to create constructs of varying thickness or collagen concentration depending on the requirement. The mechanical strength is sufficient to withstand the manipulation that would be required for transplantation without the need for any chemical crosslinking that may have deleterious effects on the behaviour of cells on the surface or after transplantation. This was demonstrated by the successful loading and delivery of RAFT to an *ex vivo* porcine eye using a clinical insertion device. Finally, one of the unfavourable aspects of the DMEK procedure is that the isolated membrane is prone to curling, making the tissue difficult to handle even by experienced surgeons and so often leads to cell loss. RAFT differs significantly in this area, showing no evidence of the spontaneous curling that can lead to cell damage, allowing easy handling during the transplantation procedure.

### Conclusions

Human corneal endothelial cells cultured on RAFT retain their endothelial characteristics on a biomaterial that has many desirable physical properties allowing easy handling. This method provides expanded human corneal endothelial cells with a suitable substrate for transplantation so that one donor cornea could potentially treat multiple patients requiring endothelial replacement.
